# The Effect of Task-Oriented Basketball Training on Motor Skill-Related Fitness in Children with Developmental Coordination Disorder

**DOI:** 10.3390/sports13030062

**Published:** 2025-02-20

**Authors:** Faiçal Farhat, Achraf Ammar, Nourhen Mezghani, Mohamed Moncef Kammoun, Khaled Trabelsi, Adnene Gharbi, Lassad Sallemi, Haithem Rebai, Wassim Moalla, Bouwien Smits-Engelsman

**Affiliations:** 1 High Institute of Sport and Physical Education of Gafsa, University of Gafsa, Gafsa 2112, Tunisia; faical.farhat@isseps.usf.tn; 2 Research Laboratory ‘NeuroPédiatrie’ (LR19ES15), Sfax Medical School, University of Sfax, Sfax 3000, Tunisia; 3 Department of Training and Movement Science, Institute of Sport Science, Johannes-Gutenberg-University Mainz, 55122 Mainz, Germany; 4 Research Laboratory, Molecular Bases of Human Pathology (LR19ES13), Faculty of Medicine of Sfax, University of Sfax, Sfax 3000, Tunisia; 5 Interdisciplinary Laboratory in Neurosciences, Physiology and Psychology: Physical Activity, Health and Learning (LINP2), UFR STAPS, Paris Nanterre University, 92000 Nanterre, France; 6 Department of Sport Sciences, College of Education, Taif University, Taif 21944, Saudi Arabia; nsmezghanni@tu.edu.sa; 7 High Institute of Sport and Physical Education of Sfax, University of Sfax, Sfax 3000, Tunisia; moncef.kammoun@isseps.usf.tn (M.M.K.); khaled.trabelsi@isseps.usf.tn (K.T.); adnenegharbi@yahoo.fr (A.G.); assad.sellami@isseps.usf.tn (L.S.); haithem.rebai@isseps.usf.tn (H.R.); wassim.moalla@isseps.usf.tn (W.M.); 8 Research Laboratory: Education, Motricity, Sport and Health (EM2S, LR19JS01), High Institute of Sport and Physical Education of Sfax, University of Sfax, Sfax 3000, Tunisia; 9 Department of Movement Sciences and Sports Training, School of Sport Science, The University of Jordan, Amman 11942, Jordan; 10 Physical Activity, Sport and Health Research Unit, National Observatory of Sport, Tunis 1003, Tunisia; 11 Sports Performance Optimization Research Laboratory (LR09SEP01), National Center for Sports Medicine and Science (CNMSS), Tunis 1003, Tunisia; 12 Physical Activity, Sport and Recreation (PhASRec), Faculty Health Sciences, North-West University, Potchefstroom 2531, South Africa; bouwiensmits@hotmail.com

**Keywords:** developmental coordination disorder, task-oriented training, motor skills, fitness, physical education

## Abstract

Developmental Coordination Disorder (DCD) is one of the most prevalent neurodevelopmental disorders in childhood. DCD is classified as a motor learning deficit because it interferes with the ability to learn and automate movement skills. There is a lack of information on how these children acquire complex motor skills relevant to their daily recreational or sports activities. Evidence to guide physical trainers, educators, and health professionals to select an effective type of training to improve physical fitness for children with poor motor coordination is scarce. The purpose of this study was to analyze the effect of an 8-week task-oriented basketball training program on motor coordination and motor skill-related fitness for DCD children in the school context. Motor performance and motor skill-related fitness were evaluated before and after the intervention using the Movement Assessment Battery for Children-2 (MABC-2) and Performance and Fitness Test Battery (PERF-FIT). A total of 52 children with DCD aged 8 to 9 were invited to join the intervention. Parents of 18 children accepted for their child to participate in the training program. In the remaining children, 20 identified as the most similar based on the diagnostic criteria for DCD (DSM-5) and anthropometric features (age, BMI) and were asked to participate as the usual care group. The difference in improvement on the MABC-2 and the PERF-FIT between the two groups on the two test occasions was compared using Mann–Whitney U tests. Within-group pre-post comparison on these test items was performed using the Wilcoxon signed rank test. Significant differences in all performance scores were found in favor of the training group. Post-hoc analysis revealed that the DCD training group improved significantly on MABC-2 total and subscores (*p* < 0.001) and on all PERF-FIT items (*p* < 0.001). No significant changes were found on any of the test items in the DCD usual care group. Group-based training in a more natural environment (playing games with peers in school) might help children with DCD as an adjunct to or before individual therapy. Based on our findings, we believe it is possible to work in large groups (*n* = 18), led by trained physical education teachers and special educators, to lessen the impact of motor coordination and physical fitness problems in children with neurodevelopmental disorders so that they can participate more easily in active games. Results of the usual care group showed that extra instruction and practice are needed for children with DCD.

## 1. Introduction

Motor competence, defined as the ability to perform a wide range of motor skills with precision, control, and coordination, is a prerequisite for enjoyable and successful participation in leisure and sports activities from childhood into adulthood [1]. It lays the foundation for a lifetime of physical activity and overall well-being [2]. Not all children develop the level of motor skill proficiency expected for their age and experience [3]. DCD is defined by a marked impairment in the ability to acquire age-appropriate motor skills, which interferes significantly with activities of daily living [4,5]. DCD is one of the most common childhood development disorders, with considerable consequences on many aspects of daily life [6]. Nonetheless, DCD is largely underrecognized by professionals in healthcare and education [7]. Although motor difficulties are core to DCD diagnosis, these limitations often have a more generalized impact on development as well as academic achievement, interpersonal relationships, poor physical health and fitness [8,9], and overall well-being [10,11]. Importantly, the motor difficulties associated with DCD limit children’s participation in active play and organized sports [12,13], predisposing them to a sedentary lifestyle [8,12]. This inactivity, in turn, negatively impacts their physical fitness [14,15] and overall health [16,17]. Children with DCD typically exhibit lower levels of aerobic and anaerobic endurance, muscular strength, and explosive power, which further restricts their ability to engage in activities requiring running, hopping, or jumping [18,19]. These fitness deficits, compounded by poor motor control, put children with DCD at higher risk for noncommunicable diseases later in life [20,21]. Given these challenges, assessing and addressing both physical activity and fitness should be integral to the management of DCD [22].

DCD is also classified as a motor learning disorder because it interferes with skill acquisition and automatization [23,24]. These challenges are particularly evident in complex tasks such as throwing and catching [24,25], which heavily rely on anticipation and action–perception coupling, known to be poor in DCD [26]. The difficulty to generalize motor skills across contexts underscores the importance of interventions that promote skill transfer. Motor learning theories, which focus on how skills acquired in one domain can be transferred to other tasks, offer a theoretical framework that could guide physical educators and sports coaches to identify more effective training strategies for children with motor coordination and fitness challenges [27]. One of the key issues in investigating skill transfer in children with DCD is understanding the mechanisms through which motor skills generalize across contexts. Some research suggests that variability in practice conditions can enhance motor learning and adaptability [28], while other studies do not confirm these findings in this population [29]. Furthermore, examining the impact of task complexity and motor skill type on transferability could provide important insights into how motor abilities learned in sports settings could affect functional performance in other domains [30]. Given that balance, coordination, and timing are central to both sports and everyday activities, understanding the transfer of these abilities in children with DCD is essential for advancing therapeutic strategies.

Given these challenges, task-oriented training has emerged as a promising approach that can improve motor competence as well as fitness [4]. Task-oriented approaches tend to focus on motor performance, i.e., on learning particular motor skills, with attention to specific aspects of task performance that are causing the child’s difficulty. Thus, the ability to transfer skills learned to other situations or contexts is an essential characteristic of this approach. These approaches yield strong effects in improving motor performance in DCD and are the recommended intervention to address deficits in motor performance and reduce activity limitations [31]. The approach aligns with ecological systems theory, which emphasizes the interaction between internal constraints, task demands, and environmental factors shaping motor behavior [32]. Motor performance shown by the child is determined by the dynamic interaction between the internal constraints of the child (like a neurodevelopmental disorder or DCD), a given task (scoring in a 3-by-3 basketball game), and environmental constraints (weather, surface, audience). Task-oriented training is usually given by physical therapists in a one-on-one setting or in a small group setting [4]. However, this is not the most natural setting for a child to learn and enjoy motor activities. Therefore, we developed a group-based training delivered by a physical education (PE) teacher together with a special educator at the school of the children. Moreover, to learn a skill, children with motor coordination problems must practice a lot and have fun to remain participating. Therefore, enjoyment and experiencing success in a peer group are important factors.

Basketball is a popular sport in Tunisia and part of the PE curriculum. For after-school activities, parents prefer their children to play basketball instead of football or handball. However, basketball is a challenging game that requires and trains speed, agility, and strength, as well as ball and visuomotor skills [33]. Many motor abilities, such as intercepting a ball at the proper time or improving landing biomechanics after a jump, must develop to be able to play the game successfully and to avoid injuries. Multi-limb coordination, temporal and spatial control, and dynamic balance and agility are required for playing the game, which all need to be adapted regularly to the position of the ball and the anticipated position of the fellow or opponent players. In this manner, information from cognitive, perceptual, and motor systems will be combined to ensure the highest potential motor performance [34]. Basketball challenges participants to respond to unpredictable and frequent environmental changes during the game [35]. As a result, basketball training will engage children in quickly examining a variety of possibilities to select a preferred strategy and predict where moving objects (ball) or people (teammates) will be. They will also learn to switch between technical actions when necessary [36]. Thus, basketball is a cognitively challenging game that needs rule comprehension, peer competition, and technical movement memory, all of which necessitate executive functions [37]. When rules are adapted, basketball can be an enjoyable game at an easier level, for instance, by adapting the size of the field, the height of the basket, and the number of players. Task differentiation to make the task (a lot) easier, is one of the principles in task-oriented training and is a basic skill for most PE teachers. In a recent study, Zolghadr et al. [38] showed that the use of mini-basketball training could improve the global motor coordination of children with intellectual disabilities and DCD.

In sum, DCD children participate less in physical activities than their peers, particularly in team sports, due to motor difficulties [4,39,40]. It is, therefore, critical to identify children who have motor difficulties needed for sports activities at a young age. Early detection can lead to early intervention, which aims to enhance involvement in sports and playground activities while also preventing later health concerns [41].

To improve their motor skills, children with DCD are frequently referred for task-oriented interventions by a pediatric physical or occupational therapist. Task-oriented approaches place a premium on enhancing skill acquisition and performance in functional activities in the actual context, thereby ensuring action–perception coupling and transfer. For the task-oriented approaches, individual and group programs are two effective modes of teaching motor skills to DCD children [4,42].

We hypothesized that a task-oriented basketball training program would lead to significant improvements in motor skills and motor skill-related fitness, such as strength, power, and agility, in children with DCD. Additionally, we aimed to assess the transfer of skills learned in the training to other motor activities. Unlike previous studies, which primarily involved small groups supervised by healthcare professionals, our study evaluated the effectiveness of a large-group intervention led by trained PE teachers. This research is particularly relevant for informing educators and sports coaches on how to design inclusive and effective training programs for children with motor coordination challenges.

## 2. Materials and Methods

### 2.1. Procedure

The protocol and study design were approved by the Ministry of Education and the Ethical Committee of the University Hospital Sfax, Tunisia (CPP SUD No. 0301/2021), in accordance with the World Medical Association’s Code of Ethics (Declaration of Helsinki, 1964 and Declaration of Tokyo, 1975, as revised in 1983). This study was registered retrospectively in the Pan African Clinical Trials Registry (PACTR202311749369035).

**Figure 1:**
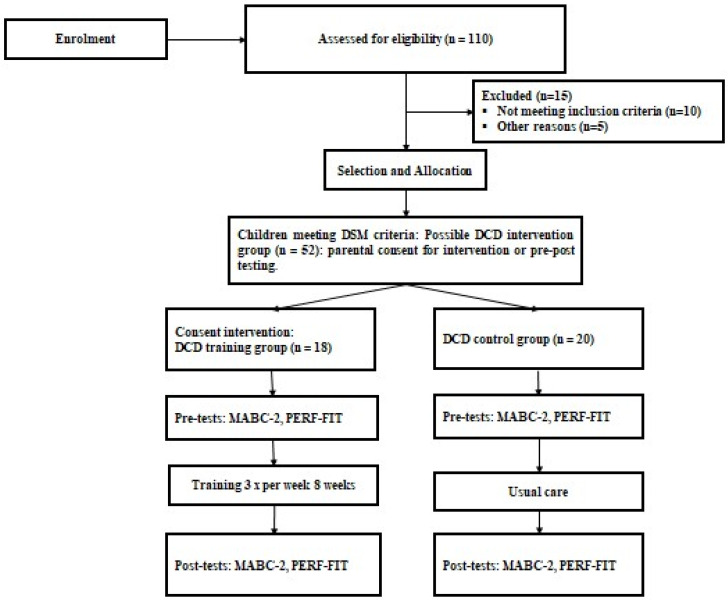
CONSORT flow diagram of the recruitment process and data collection.

The steps in the recruitment of participants for the training program are shown in the flowchart (Figure 1). Children were selected from five different schools. The assessments and training were performed after the parents completed the written consent forms. To select the children with DCD, we used the operationalized Diagnostic and Statistical Manual of Mental Disorders DSM-5 DCD criteria [3]. The person responsible for selecting the children with DCD had extensive experience in the application of DSM-5 criteria, having worked with children with developmental disorders for several years. Additionally, the selection process was conducted under the supervision of a pediatric neurologist with specialized training in DCD. To ensure accurate diagnosis, each child underwent a comprehensive assessment that included observational assessments (MABC-2 test) and clinical interviews, and standardized questionnaires (DCD-Q) for the parents, all aligned with DSM-5 criteria for DCD. This rigorous approach was used to confirm that each child met the specific characteristics of developmental coordination disorder. The recruitment process for the study began with estimating the required sample size to ensure sufficient statistical power. After determining the sample size needed, 52 children diagnosed with DCD were initially invited for participation. Parents of 18 children accepted for their child to participate in the training program (DCD training group). The other parents agreed to participate in the evaluation pre- and post-training, as they stated that their child was unable to participate in the intervention sessions three times a week for eight weeks. From this list of children with DCD not included in the training group, those identified as the most similar, based on the diagnostic criteria for DCD (DSM-5) and anthropometric features (age, BMI), were asked to participate as the usual care group. Through this procedure, 20 children were selected who matched best to the DCD training group.

All the children completed the assent forms. The selected 38 children were tested twice, at baseline and the end of the training period. All pre-tests were administered a maximum of one week apart. These pre- and post-tests were administered to the children by five trained sports teachers, a physical education teacher, and an occupational therapist. They were trained in the test protocol but were unaware whether children would receive or had received the intervention.

Movement Assessment Battery for Children-2 (MABC-2) was administered individually in a quiet room, and the Performance and Fitness Test Battery (PERF-FIT) was performed in the school sports hall during a physical education class. Before scoring, each test item was explained to the child, demonstrated, and practiced according to the manuals.

### 2.2. Participants

A total of 38 DCD children aged 8 to 9 were classified using the four DSM-5 criteria [1]. There were 13 boys and 5 girls in the DCD training group and 14 boys and 6 girls in the DCD usual care group. All children in the study had a MABC-2 score at or below the 16th percentile (Criterion A) and were identified based on the DCDDaily-Q [43]. The DCDDaily-Q is a useful tool for assessing the impact of DCD on a child’s daily life (Criterion B). It helps to identify specific motor difficulties that children with DCD may encounter in everyday activities such as dressing, eating, or school tasks. By collecting information from parents or caregivers, the DCDDaily-Q provides insights into how these challenges affect a child’s daily functioning, which can guide intervention strategies and help track progress over time [4]. Their parents noted difficulty throughout the early developmental period (Criterion C), and there were no reported medical conditions or comorbidity known to interfere with motor abilities in the parent questionnaire. The teacher confirmed the absence of intellectual or cognitive disability (Criterion D).

Sample size was estimated based on the expected effect size of the training versus the usual care group on the primary outcome (PERF-FIT). We calculated the sample size of this study through G* Power v3.1.9.2 software (Germany), using a previous study that also used the PERF-FIT [44] as a reference. We assumed a power of 0.80, an alpha of 0.05, and an effect size of 0.85. The total minimal sample size was estimated to be 19 per group to detect meaningful differences in motor fitness outcomes of this study.

### 2.3. Measurements

#### 2.3.1. The Movement Assessment Battery for Children-2 (MABC-2)

The MABC-2 test was used to assess motor coordination [45]. Because the children were between 7 and 10 years of age, they completed MABC-2 age band 2. The MABC-2 test consists of eight items that are evaluated in three different components: manual dexterity, aiming and catching, and balance. Total standard scores can be converted to percentile scores, with higher scores suggesting better performance. Scores above the 16th percentile indicate motor performance in the normal range. Scores between the 9th and 16th percentiles are considered “at risk of motor difficulty”, and scores at or below the 5th percentile are considered to indicate “motor coordination problems”. With ICC values ranging from 0.92 to 0.98, the MABC-2 test has shown good validity and test–retest reliability [45]. The MABC-2 was chosen for this study to confirm criterion A of the DSM classification of the children and to make the results of this study comparable to other studies, as the MABC-2 is the most frequently reported outcome in intervention studies [46].

#### 2.3.2. Performance and Fitness Test Battery (PERF-FIT)

The PERF-FIT is a functional assessment of motor skill-related fitness for children aged 6 to 12, developed to measure skills needed in active play [47]. The PERF-FIT consists of two distinct components: motor performance and power and agility. The motor performance subscale consists of a skill item series for bouncing and catching, throwing and catching, static and dynamic balance, and jumping and hopping. Five test items constitute the power and agility subscale: running (agility), stepping (agility), side jump (muscular endurance), standing long jump (muscular power of the legs), and overhead throw (muscular power of the arms). The PERF-FIT battery is a reliable and valid test for children aged 6 to 12, with high content validity (content validity index ranging from 0.86 to 1.00), excellent structural validity, excellent inter-rater reliability (ICC. 0.99), and good test–retest reliability (ICC. ≥ 0.80) [48]. The PERF-FIT was chosen as the primary outcome because it covers the constructs the training focused on (motor skill-related fitness) and has shown to be suitable for this context.

### 2.4. Training Program

The usual care group consisted of children with DCD who maintained their standard classroom activities and PE classes throughout the trial, while the training group took part in an 8-week after-school training program. The task-oriented motor skill training program focused on basketball-related skills, which was administered for eight weeks, three times a week in 60 min sessions to the DCD training group. Two coaches trained all 18 children as they exercised in one group. The coaches stayed as close to the final tasks and context when adapting the basketball exercises to the level of the children to enhance transfer to basketball participation. The program included a 10 min warm-up focused on activities designed to prepare their bodies for the main activity, 30–35 min fitness and agility training using basketball drills, 10 min of variable training shooting basketballs from various distances, a 15 min reduced basketball game 3 versus 3, and a 5 min cooldown. The two coaches in charge of the DCD training group (physical education teacher and special education teacher) introduced and explained the basketball game rules. The fundamental gross motor abilities (jumping, throwing, catching, and running with directional changes) that are regarded to be prerequisites for basketball sports team abilities were demonstrated to and practiced by the children. The task difficulty was raised in a structured manner over the intervention period.

Table 1 summarizes the group training activities. The training included task and environmental constraint adaptations, as well as motor learning and ecological principles of skill development [49]. Adapting tasks to the child’s ability level will increase positive involvement. The motor tasks were modified as the training progressed to create a balance between an adequate challenge to the child’s motor ability and successful task completion. Attendance to the training sessions was 90%.

### 2.5. Statistical Procedure

The Shapiro–Wilks test was used to determine the normal distribution of all variables. Based on the findings and the small sample size, non-parametric testing was used. As the quasi-randomization does not guarantee to equally balance all child characteristics across groups, data on child characteristics were used to evaluate the degree of comparability of these factors across the 2 groups. Demographic characteristics and motor skill levels between groups were compared using a Mann–Whitney U test. The training effect was assessed by comparing the difference on the MABC-2 and PERF-FIT between pre- and post-test performance of the two groups (DCD training group, DCD usual care group). Additionally, pairwise comparison (related-samples Wilcoxon signed rank test) was used to determine if there were significant within-group changes between the two test moments. Effect size r was calculated as Z statistic divided by the square root of the sample size (N) (Z/√N). Cohen (1988) gives the following intervals for r effect sizes: 0.1 to 0.3: small effect; 0.3 to 0.5: medium effect; 0.5 and above: strong effect. The individual change between pre- and post-test was evaluated by the percentage of children who improved more than the Minimal Important Difference and Smallest Detectable Change on the items of the MABC-2 [50] and PERF-FIT [48], respectively. The statistical package for social sciences software (SPSS, version 29.0, SPSS Inc., Chicago, IL, USA) was used for all statistical analyses.

## 3. Results

### 3.1. Participants

Table 2 summarizes the demographic characteristics of the DCD training group (*n* = 18) and the DCD usual care group (*n* = 20). At baseline, the two groups had comparable mean age, height, and BMI. Importantly, no differences in the motor tests (MABC-2, PERF-FIT) were identified between the two DCD groups, indicating that the two groups were comparable in the important characteristics when entering the study at baseline.

### 3.2. Comparison Between Type of Intervention on Pre- and Post-Data

The training effect was assessed by comparing the difference in pre- and post-test data for the two groups (DCD training group, DCD usual care group). The Mann–Whitney U test indicated a significantly better result for the training group compared to the usual care group on all items measured (Table 3). Post hoc pre-post analysis per group confirmed that the training group significantly improved on all items measured by the MABC-2 and the PERF-FIT with large effect sizes (Table 4). Table 4 shows the medians (min-max) and statistics for the pairwise comparisons at pre- and post-test for all outcomes for the DCD training group and DCD usual care group.

#### 3.2.1. MABC-2

Group statistics showed that the DCD training group improved significantly more than the usual care group (Table 3 and Table 4). In addition to the group comparison, we also examined individual change in classification and the percentage of children who improved more than the Minimal Important Difference (MID). MID, which is the smallest change in score that clinicians perceive as important for the MABC-2, has been reported to be 2.36 [50]. This would mean that 44% of the intervention children improved more than the MID on the total score. After training, 11 children in the training group were classified as scoring in the normal range (≥25th) and 7 in the at-risk range. All children in the usual care group still scored at risk (*n* = 8) or in the impaired range (*n* = 12).

#### 3.2.2. PERF-FIT Motor Skill Performance

There was a positive significant difference (*p* < 0.001) of the effect of training on all the PERF-FIT motor skill items (Table 3 and Table 4). The individual change was evaluated using the Smallest Detectable Change (SDC) [48]. The percentage of children who improved more than the SDC was for jumping 89%, hopping right 89%, hopping left 78%, bouncing and catching 22%, throwing and catching 22%, and static 44% and dynamic balance 66%. None of the children in the usual care group changed more than the SDC.

#### 3.2.3. PERF-FIT Agility and Power

There was a positive significant difference (*p* < 0.001) in the effect of training on the PERF-FIT agility (running, stepping) and power (side jump, standing long jump, and overhead throw) items (Table 3 and Table 4). The percentage of children who improved more than the SDC was for running 89%, stepping 78%, side jump 61%, standing long jump 72%, and overhead throw 44%). None of the children in the usual care group changed more than the SDC.

## 4. Discussion

The current study aimed to determine the effect of a task-oriented training program, based on the theoretical foundation that motor performance is the result of interactions between characteristics of child, task, and environment. In their guideline, the European Academy for Childhood Disability (EACD) suggests that task-oriented approaches are the recommended intervention strategies for children with DCD and currently have the best cost-benefit [51]. Yet the current knowledge regarding the effects on motor performance and fitness of task-oriented interventions delivered in a group format to children with DCD is limited. Developmental coordination disorder (DCD) is categorized as a motor learning deficit. Thus, this group of children needs extra opportunities to explore and learn motor skills. Therefore, an 8-week training program on basketball-related skills was conducted, and it was assessed whether the children improved in motor activities (e.g., throwing, catching, jumping) and skill-related fitness (power and agility). As expected, the usual care group (DCD) showed no significant change in any of the outcomes after nearly three months without extra training. This group only received normal PE classes and unstructured play at recess and after school. Normal development and these activities did not lead to improved performance. The fact that the usual care group showed no change in any of the outcome variables demonstrated that usual care did not help the children improve their motor skills. The usual care group results also emphasized stable performance on the PERF-FIT and MABC-2 and no obvious learning effect after this interval.

Typically, schools provide support for children with challenges in acquiring academic skills, such as reading, writing, comprehension, and mathematical abilities. It is, however, equally important to address motor learning difficulties, particularly for children with DCD. These children not only need specialized programs for motor skill development but also require approaches that promote the transfer of these skills to broader life contexts. The concept of skill transfer has been explored in typically developing children, showing that motor skills gained through sport can be applied to everyday activities [52]. For example, aspects such as balance and coordination learned through sports like basketball or gymnastics can enhance children’s performance in tasks like walking or riding a bicycle [53]. However, for children with DCD, the process may be more complicated due to underlying difficulties in sensory processing and motor planning, which can hinder the generalization of skills from structured sports contexts to other activities [49].

The structured program tested in this study demonstrated substantial enhancements in specific motor skills. Tailoring extra motor activities to the needs of children with DCD could be provided within the school context. In case the children do not catch up considerably or if the motor challenges are large, the child could be referred to additional one-on-one intervention with a physio or occupational therapist.

After an 8-week training program of 3 h per week, the children in the training group showed a large increase (r-0.83–0.89) in motor skills, agility, and power. Our data showed specific basketball-related training effects; DCD children improved in bouncing, throwing and catching, jumping, and overhand throwing. These findings support earlier research on the effectiveness of task-oriented intervention. Bonney et al. [54] demonstrated the efficiency of these approaches in motor performance increase in female adolescents with DCD. After task-oriented functional training, significant improvements were found in muscular strength (strength of the knee extensors, ankle plantar flexors and dorsiflexors) and running performance (anaerobic sprint tasks: 10 × 5 m straight and slalom tests) [54]. According to our findings, task-oriented basketball-based training can potentially decrease motor coordination and physical fitness difficulties in DCD populations, even if given in a large group. Ferguson et al. [55] showed that group-based Neuromotor Task Training (NTT), a specific form of oriented training, can be used effectively to treat children with DCD in areas of resource constraints, such as low-income schools. It has also been found that group-based training produced similar gains in motor performance to individual-based training, and group-based training may be the preferred treatment option due to the associated cost savings [56]. Notably, the common group size in intervention studies for children with motor difficulties lies around 3–11 [49,55,57]. For example, children with DCD were divided into groups of three or four based on shared occupational performance problems in the study by Thornton et al. [58]. The group size was four or five based on motor skill level and gender in the Cacola et al. study [59]. So, the group size of eighteen with two coaches in the current intervention study may be a good use of resources. Also, the fact that the training was given at their school is seen as an advantage and may enhance the daily use of the newly learned skills.

Although it has previously been demonstrated that group-based training achieved comparable gains in motor performance to individual-based training, DCD is a very heterogeneous condition with many comorbidities such as ADHD, learning disabilities, anxiety, and speech or language disorders [60]. This was one of the reasons why group size had to be very small (groups of four children with two supervisors) in children with combined motor, attentional, and learning disabilities in a nine-week task-oriented program [61] that was run in a school for special education. Hence, group size needs to be determined based on the joint goals of children for the intervention, age, and, importantly, the presence of comorbid behavioral, attention, or cognitive problems.

Moreover, the current study’s findings revealed positive effects of not specifically trained aspects (like picking up a can off the floor while keeping balance on one leg). However, several activities in our training involved dynamic balance, such as restoring balance after jumping and quick changes of direction. These findings are consistent with the results of Cacola et al. [59], who indicated that both group-based task-oriented training and goal-oriented training were effective in increasing DCD children’s balance and overall motor abilities. Our study also showed that children were able to adapt the trained skill with a basketball to a new context (throwing, bouncing, catching with preferred and non-preferred hand with a tennis ball).

Remarkably, the subscore manual dexterity of the MABC-2 also showed improvements. Poor fine motor abilities are often associated with visual perception deficits and attention deficits [62,63,64], which might have been improved by the training. Recently, Hashemi et al. [65] showed that children with DCD improved their visual perception after 8 weeks of Wii Fit training, and Jelsma et al. [66] found improvements in sustained attention after 5 weeks of Wii Fit training. Hammond et al. [67] also reported improvements in fine motor precision and visuomotor integration among children with DCD after Wii Fit balance games, which require sustained focus and response selection based on actions on the screen. The integration between response selection or inhibition and task switching based on acquired information about changing conditions is trained within the motor task using a task-oriented approach [66]. The potential changes in visuomotor integration, eye movements, and divided and sustained attention in the children participating in our study were not evaluated. Because many aspects of skill behavior were trained at the same time in the current study, it was difficult to determine which aspects caused the transfer effects.

To the best of our knowledge, the current trial was the first to show large effect sizes, indicating significant improvements, after a basketball-based intervention in DCD children. The training program was designed with careful consideration of the specific needs of children with DCD. Recognizing that basketball requires a significant level of coordination and skill, the program was structured to address these challenges progressively. It was assumed that the children had no prior experience with the sport, so the initial stages focused on basic motor aspects, such as hand–eye coordination and body movements, before gradually introducing more complex basketball techniques. The progression of the program was tailored to ensure that the children could build confidence and competence step by step, starting with fundamental tasks and advancing as their abilities improved. Throughout the intervention, the emphasis was placed on creating an encouraging environment to foster both physical and psychological growth, helping the children overcome the difficulties associated with their condition while learning the sport.

The children with DCD were clearly able to learn skills and also able to transfer what was trained to other contexts as confirmed by significant changes on all items of the PERF-FIT. By bouncing, throwing, and catching a basketball, they also improved on bouncing, throwing, and catching a tennis ball. This corroborates earlier results, which showed that children with DCD performed better on both trained and non-trained tasks such as ball, balance, and agility after 10 weeks of training [68]. Our results also align with a recent study by Amato et al. [69], which found that typically developing children who play basketball perform better on manual dexterity tasks compared to those involved in other sports such as soccer, volleyball, dance, gymnastics, karate, and kickboxing. Their study conclusions suggested that playing basketball could contribute to improving manual dexterity by promoting the development of complex motor skills through activities like dribbling, passing, and shooting. Moreover, as the task becomes more complex, prediction and adaptation become more difficult, and processes of executive functioning are needed, such as attention, working memory, planning, and problem solving.

Basketball practice requires the fast processing of visuospatial information, which may transfer to other motor tasks [68]. Children use their visuospatial working memory when they learn how to dribble around adversaries during the game. Movement repetition and adaptation to game situations in basketball practice permit children to refine and learn motor skills, such as intercepting a ball, controlling precision, coordination, and aiming. These acquired and improved qualities may have allowed the improvement in comparable activities using tennis balls after the training.

It has been shown earlier that children with DCD can learn in a more complicated sport-based group training such as soccer [70] and trampoline jumping [71,72]. Our findings demonstrated that children with DCD can learn a motor skill if given enough practice and can benefit from motor learning-based instruction, even if certain adaptations are required. Basketball challenges participants to respond to unpredictable and frequent environmental changes during the game [35]. As a result, basketball training will engage children in quickly examining different possibilities to select a preferred strategy and predict where moving objects (ball) or people (teammates) will be. They will also learn to switch between technical actions when necessary [36]. Basketball is a cognitively challenging game that needs rule comprehension, peer competition, and technical movement memory, all of which necessitate executive functions [37].

### 4.1. Limitations and Perspectives

The method of allocation used in this study falls short of randomization, which increases the risk of bias. Some baseline confounding might occur because parents of the included children were willing to let their children participate three times a week in the training while the usual care group parents only agreed to pre-post testing sessions. However, it is unlikely that these factors can explain the magnitude of the current changes. No risk of bias due to deviation from the intended intervention or missing data occurred. Additionally, the testers were blinded to the group assignment. Retention tests were not administered; thus, it is impossible to predict if the duration of the training was long enough to benefit children with DCD in the long run. Another limitation of the study is the absence of a third comparison group based on another sport with the same frequency and duration as basketball. Moreover, we did not register potential confounding factors, such as the level of involvement in physical activities outside of school. These factors could influence the outcomes, and without adjusting for them, it becomes challenging to accurately attribute the observed effects to the basketball training itself.

To address these limitations, we proposed that future research be designed as randomized controlled trials that evaluate not only pre- and post-intervention but also retention. Additionally, measuring visuomotor integration, eye movements, attention, and executive function might help find changes in underlying processes that might explain transfer effects.

### 4.2. Practical Implications

To effectively support children with Developmental Coordination Disorder (DCD), intervention programs should implement targeted motor skill training. The following practical recommendations can help maximize the benefits of such interventions:

1. Integrate structured motor skill programs into physical education or extracurricular activities to address motor challenges alongside academic learning [22].
2. Implement sport-based, task-oriented training, such as basketball, to improve specific motor skills and enhance coordination and agility in everyday tasks.
3. Adjust group sizes based on children’s motor skill levels and comorbidities, ensuring that children with more complex needs receive adequate support. In those cases, smaller groups may be necessary for effective intervention [4].
4. Incorporate activities that promote skill transfer to real-life contexts, helping children apply learned motor skills to daily functional tasks.
5. Recognize the cognitive benefits of motor training, as it can enhance attention, visual perception, and other cognitive functions alongside physical skill development [66].

## 5. Conclusions

The findings of the present study demonstrated that task-oriented basketball training significantly improved motor skills, agility, and power in children with DCD. The intervention also led to improvements in untrained motor tasks, such as balance and manual dexterity. Importantly, regular PE and recess activities are clearly not enough for children with DCD to develop new skills. Whether the improvement of motor skills in some of these children was just a matter of more practice or dependent on the specific training principles used in this study has yet to be evaluated in a direct comparison study. However, as shown in the literature and our study without intervention, children with DCD do not improve their motor skills, whereas they do improve after task-oriented intervention.

In summary, we have demonstrated that group-based motor skill training in the school setting is feasible and increases motor coordination and motor skill-related fitness in children with DCD. Additionally, there was some improvement in non-trained tasks. Usual care, consisting of physical education classes and regular physical activities, did not elicit improvements in any of the outcomes in the usual care DCD group, showing that extra instruction and practice are needed for this group of children.

## Figures and Tables

**Table 1:** Task-oriented training program (8 weeks).

Skills	Exercises	Materials
Week 1–2		
Coordination	Dribbling from standing, sitting down and getting up without losing the ball.	18 balls
Pass and catch	Spiky ball, pass and catch against an elastic net.	2 groups, 18 balls
Reaction speed	Running forward, backward, and sideways upon trainer’s signal.	3 groups
Strength-coordination	American jumping on a ladder, dribbling in slalom, and pass the ball to their partner.	2 ladders, 12 plastic sticks
Shoot	Lineup behind the cone and shooting from different spots and distances.	6 groups, 6 balls
Rule, defense-attack	Reduced game 3 against 3.	3 mini grounds
Cool-down stretches	Active and passive flexibility.	Body only
Week 3–4		
Pass and catch	The child in front passes the ball alternately for 2 partners (moving along line).	6 groups, 6 balls
Shoot-precision	Standing behind a cone, throw a tennis or basketball into a bucket from a 2–3 m distance.	6 groups, 6 tennis balls, 6 basketballs
Pass and catch	Spiky ball, pass and catch against an elastic net at his partner’s chest level.	2 groups, 18 balls
Strength-coordination	Jumping forwards, backward, left and right, sprinting upon trainer’s signal with and without dribbling.	3 groups
Shoot	Line up behind the cone and shoot from different spots and distances.	6 groups, 6 balls
Rule, defense-attack	Reduced game 3 against 3.	3 mini grounds
Cool-down stretches	Active and passive flexibility.	Body only
Week 5–6		
Pass and catch	Throwing a tennis or basketball, walling and catching.	18 tennis balls, 18 basketballs
Coordination	Dribbling in a ladder.	2 ladders
Strength-coordination	Jumping the hurdles without losing the ball.	12 hurdles, 2 groups
Strength	Running forward, touch the cone (color shown), and running backward.	3 groups, 9 plastic sticks
Shoot-precision	Line up behind the cone and shoot from different spots and distances.	6 groups, 6 balls
Rule, defense-attack	Reduced game 3 against 3.	3 mini grounds
Cool-down stretches	Active and passive flexibility	Body only
Week 7–8		
Reaction speed	The teacher throws the ball up, on the rebound the child must catch it before his opponent.	One against one
Strength-coordination	Jumping the hurdles, skipping in the hoops with and without dribbling.	12 hurdles, 12 hoops
Coordination	Face to face, dribbling a basketball with one hand, passing a tennis ball with the other hand.	18 tennis balls, 18 basketballs
Coordination	Dribbling, putting the cone in the hoop and returning to starting position.	18 balls, 6 cones, 6 hoops
Shoot-precision	Line up behind the cone and shoot from different spots and distances.	6 groups, 6 balls
Rule, defense-attack	Reduced game 3 against 3.	3 mini grounds
Cool-down stretches	Active and passive flexibility.	Body only

Mini grounds: smaller areas or spaces used for specific activities or tasks. Ball Circumference: 27.5″.

**Table 2:** Demographic data of children with and without DCD.

	DCD Training Group (*n* = 18)			DCD Non-Training Group (*n* = 20)				
	Median	Minimum	Maximum	Median	Minimum	Maximum	z-Scores	*p*-Value
Age (years)	8.6	8	8.9	8.5	8.1	9.9	−0.117	0.907
Height (m)	1.35	1.30	1.44	1.33	1.27	1.45	−0.192	0.848
Body mass (kg)	33.50	30	40	31	27	38	−2.108	0.035
BMI (kg m^−2^)	18.45	16	22	17.64	15	20	−1.463	0.144

DCD, developmental coordination disorder.

**Table 3:** Statistics for the differences between pre- and post-tests between groups on MABC-2 test and PERF-FIT.

	z-Value	*p*-Value
MABC-2		
Total Standard Scores	−5.256	<0.001
Manual Dexterity (SS)	−4.843	<0.001
Aiming and Catching (SS)	−4.071	<0.001
Balance (SS)	−3.029	<0.01
PERF-FIT		
Jumping (#)	−5.252	<0.001
Hopping right (#)	−5.324	<0.001
Hopping left (#)	−5.239	<0.001
Bouncing and Catching (#)	−5.029	<0.001
Throwing and Catching (#)	−5.303	<0.001
Static Balance (s)	−4.825	<0.001
Dynamic Balance (#)	−4.654	<0.001
Running (s)	−5.007	<0.001
Stepping (s)	−5.239	<0.001
Side Jump (#)	−5.042	<0.001
Long Jump (cm)	−5.196	<0.001
Overhand Throw (cm)	−4.876	<0.001

SS, standard score; s, second; #, measured in number.

**Table 4:** Pair-wise comparison (Wilcoxon) of MABC-2 and PERF-FIT (median and range) at pre- and post-test per group.

	DCD Training Group (*n* = 18)							DCD Usual Care Group (*n* = 20)					
	Median Pre	Median Post	Range Pre	Range Post	z-Score	*p*-Value	Effect size r	Median Pre	Median Post	Range Pre	Range Post	z-Score	*p*-Value
MABC-2													
Total Standard Scores	5	8	3	4	3.755	<0.001	0.89	4	4	4	4	0.816	0.414
Manual Dexterity (SS)	6	9	3	4	3.758	<0.001	0.89	6	6	5	6	−1.03	0.31
Aiming and Catching (SS)	6	8	5	4	3.647	<0.001	0.86	6	6	6	4	−0.319	0.749
Balance (SS)	6.50	8.50	4	5	3.384	<0.001	0.80	5	6	6	5	−0.183	0.855
PERF-FIT													
Jumping (#)	15	20	4	7	3.744	<0.001	0.88	15	15	3	4	0.32	0.32
Hopping right (#)	9	17	3	7	3.745	<0.001	0.88	9	9.50	4	4	0.471	0.637
Hopping left (#)	8	15	2	9	3.736	<0.001	0.83	8.50	9	3	3	1.268	0.205
Bouncing and Catching (#)	37.50	43	4	5	3.738	<0.001	0.88	37	38	4	7	0.784	0.433
Throwing and Catching (#)	34.50	43.50	4	8	3.742	<0.001	0.88	35	35	6	10	0.599	0.61
Static Balance (s)	32.20	40.75	19	20	3.680	<0.001	0.87	33.50	32.90	10	9	−1.76	0.240
Dynamic Balance (#)	15.50	22	5	10	3.524	<0.001	0.83	15	15	7	5	−0.931	0.352
Running (s)	10.05	7.55	2	2	−3.729	<0.001	0.88	10.25	10	2	2	−0.692	0.489
Stepping (s)	18.20	14.55	2	3	−3.724	<0.001	0.88	18.10	18.05	2	3	−0.038	0.97
Side Jump (#)	15	21	3	7	3.755	<0.001	0.88	14.50	15	4	4	0.707	0.479
Long Jump (cm)	90	125	23	35	3.724	<0.001	0.88	89.50	95	20	25	1.361	0.173
Overhand Throw (cm)	200.50	235	22	45	3.520	<0.001	0.83	200	200	39	40	−1.55	0.121

MABC-2, Movement Assessment Battery for Children-2; SS, standard score; s, second; PERF-FIT, the Performance and Fitness Test; #, measured in number.

## Data Availability

The data presented in this study are available on request from the corresponding author due to privacy protection.
